# A Survey on How Ocular Surface *Demodex* Infestation Interactively Associates with Diabetes Mellitus and Dry Eye Disease

**DOI:** 10.1007/s11686-021-00382-8

**Published:** 2021-04-03

**Authors:** Chang Huang, Shuze Chen, Sheng Fu, Yingli Li, Zhenhao Li, Siqi Li, Xiaoqian Liang, Zihong Wang, Zhoucheng Wang, Yifan Chen, Qixin Deng, Guoguo Yi, Min Fu

**Affiliations:** 1 The Second Clinical School, Southern Medical University, Guangzhou, Guangdong China; 2 Department of Gastroenterology, Nanfang Hospital, Southern Medical University, Guangzhou, Guangdong China; 3 The University of South China, Hengyang, Hunan China; 4 Department of Ophthalmology, Shenzhen Hospital, Southern Medical University, 1333 Xinhu Road, Baoan District, Shenzhen, China; 5 Department of General Surgery, Nanfang Hospital, Southern Medical University, Guangzhou, Guangdong China; 6 Department of Pediatrics, Zhujiang Hospital, Southern Medical University, Guangzhou, Guangdong China; 7 Department of Ophthalmology, The Sixth Affiliated Hospital of Sun Yat-Sen University, Guangzhou, Guangdong China; 8 Department of Ophthalmology, Zhujiang Hospital, Southern Medical University, No. 253 Industrial Avenue Center, Haizhu District, Guangzhou, Guangdong China

**Keywords:** Ocular surface *Demodex* infestation, Diabetes mellitus, Dry eye disease, Cross-sectional study

## Abstract

**Purpose:**

Prevention of ocular surface (OS) Demodex infestation plays an important role in OS hygiene and variety of factors may be associated with it, in which diabetes mellitus (DM) or dry eye disease (DED) has caught the attention of most scholars. However, there has been no research on whether there was a potential interaction between DM and DED in the process of OS Demodex infestation. This cross-sectional study was implemented in Zhujiang Hospital of Southern Medical University.

**Methods:**

Ophthalmologic interviews, questionnaires, and examinations were conducted. Factors including general information, DM status, dry eye condition, etc. were collected to study the correlation of DM and DED on OS Demodex infestation.

**Results:**

After statistical analysis, we found that both DM (*P* < 0.001) and DED (*P* = 0.013 < 0.05) are closely associated with OS Demodex infestation. Compared with DED, DM has higher priority association with OS Demodex infestation, and patients with both diseases have a significant higher risk of OS Demodex infestation (*R* = 0.197, *P* < 0.001). Meanwhile, age (*R* = 0.299, *P* < 0.001) and hypertension (*P* < 0.05) were also correlated with OS Demodex infestation.

**Conclusion:**

This study provides a new evidence-based basis for clinical prevention and management of OS Demodex infestation.

## Introduction

Ocular surface (OS) *Demodex* infestation is a common disease in population [1, 2]. *Demodex* (Acariformes:Prostigmata) is one of the most common ectoparasites in humans [3]. Among many species of *Demodex*, only *Demodex folliculorum* and *Demodex brevis Akbulatova* are found in humans, including the face, cheeks, forehead, nose, and eyelids [4, 5]. Eyelid, follicular mycosis lives in ciliated sacs. Besides, short mold lives in eyelid, eyelid gland and sebaceous gland [6]. They eat skin cells, hormones and oils from hair follicles [7]. The pathogenic role of *Demodex* in ophthalmology has not been controversial [8]. For some, it is a contributing factor, for others it has no obvious symptoms. This indicates that an individual's response depends on a number of factors. Some studies have shown that *Demodex* is a non-pathogenic parasite [9]. However, the other reports suggest that OS *Demodex* infestation was a critical factor in many eye diseases [1, 10], such as nodular corneal scars, blepharitis, conjunctivitis and trichiasis. Thus, the prevention of OS *Demodex* infestation plays an important role in ocular surface hygiene. It has been found that multitudinous factors may affect OS *Demodex* infestation, such as age, diabetes mellitus (DM) [11], dry eye disease (DED) [12], and meibomian gland dysfunction (MGD) [13], etc.

DM is a metabolic disease characterized by hyperglycemia which can lead to chronic damage and dysfunction of various tissues [14]. Previous studys showed that *Demodex follicle sacs* were more common in diabetic patients than in healthy volunteers [15, 16]. Beside, DED is a disorder of the tear film, which is a highly prevalent chronic multifactorial disease, causing an itchy eyes or foreign body sensation. Many reports [17] suggest that OS *Demodex* infestation is prevalent in DED patients. It had been reported that *Demodex folliculorum* showed a high prevalence in patients with DED [17, 18]. Meanwhile, another study indicated that *Demodex* were implicated in DED syndrome [19].

To futher understand whether there was an interaction between DM and DED in the process of OS *Demodex* infestation, we designed a cross-sectional study to explore the interaction between DM and DED in OS *Demodex* infestation. Hence, we can provide an evidence-based medicine for the future prevention and treatment of ocular surface *Demodex* infestation.

## Materials and Methods

This cross-sectional study was conducted from June 2018 to October 2018 at the Zhujiang Hospital of Southern Medical University in China. The research has been registered on Chinese clinical trial registry (ChiCTR1800016357). The study was performed in accordance with principles of the Declaration of Helsinki, the International Conference on Harmonisation, Good Clinical Practice guidelines, and all applicable laws and regulations. The study protocol and one amendment to the protocol were reviewed and approved by the Zhujiang Hospital Human Experimental Committee, and all patients provided written informed consent prior to starting study treatment.

### Participants

This study recruited 255 outpatients and inpatients from Zhujiang Hospital of Southern Medical University. The exclusion criteria were as follows: recent acute complications and infestations such as diabetic ketoacidosis; history of eye surgery and trauma within 6 months; cornea contact lens wear history; elevated blood glucose caused by Type I DM and other causes; dry eye caused by rheumatic immune disease and hyperthyroidism; pregnancy, lactating female; and mentally ill patients. Ophthalmologic interviews, questionnaires and examinations were performed to all participants. Data of all participants included general information (age, sex, weight, height, and history of other diseases such heart disease), routine blood test, hypertension, etc. To facilitate the analysis of age, we divide the age into several stages: 0–9, 10–19, 20–29, 30–39, 40–49, 50–59, 60–69, 70–79, and 80–89. The outcome variables were ocular surface disease index (OSDI) symptom questionnaire, tear film break-up time, eyelid symptom score, total cholesterol, fasting blood glucose, fasting insulin, HbA1c and so on. Outcomes were cross-sectionally conducted on factors associated with OS *Demodex* infestation, including *D. folliculorum* and *D. brevis.*

### Assessment of Ocular Surface *Demodex* Infestation

Two lashes were removed from each eyelid by fine forceps under a slit-lamp microscope for each patient. The removed lashes from each eyelid were placed separately on a glass slide. OS *Demodex* detection and counting was performed by a professional technician who did not know each patient’s information. Under a light microscope, 1 drop of saline or fluorescein-containing solution was applied by a pipette to the edge of the coverslip before counting.

The diagnosis of ocular surface *Demodex* infestation is based on the 2018 expert consensus on diagnosis and treatment of Demodex blepharitis China diagnostic criteria: (1) *Demodex* in all phases are counted; (2) adult patients have a *Demodex* count of 3/3 eyelashes in any of the four eyelids; (3) less than the above criteria is suspected positive, combined with clinical manifestations; if necessary, other pathogenic microorganisms can be examined at the same time, such as bacteria and fungi. Therefore, the severity of OS *Demodex* infestation is divided into 3 level: negative, suspicious positive and positive.

### Assessment of Diabetes Mellitus

Diabetes mellitus was diagnosed according to the 2018 US ADA diagnostic criteria: (1) glycosylated hemoglobin HbA1c ≥ 6.5%. (2) Fasting blood glucose FPG ≥ 7.0 mmol/L. Fasting is defined as no calorie intake for at least 8 h. (3) In the oral glucose tolerance test, the blood glucose was ≥ 11.1 mmol/L for 2 h. (4) In patients with typical hyperglycemia or hyperglycemia crisis, the random blood glucose was ≥ 11.1 mmol/L. The duration of DM was divided into the following stages: 0 years, ≤ 5 years, ≤ 10 years, and ≥ 10 years.

### Assessment of Dry Eye Outcomes

Based on American Academy of Ophthalmology and DEWS, the dry eye patients was define as individuals who had at least one of the classical symptoms plus one or more alterations in the objective tests analyzed. They were evaluated for common symptoms of dry eye ocular discomfort including: soreness, gritty sensation, itchiness, redness, blurred vision that improves with blinking, and excessive tearing. Both the frequency of these symptoms were based on criteria proposed by the American Academy of Ophthalmology and DEWS.

To analyze the severity of DED, we divide the OSDI scores into 4 degrees: no obvious symptoms: OSDI scores ≤ 12.0. Mild: 12.00 < scores ≤ 22.0. Moderate: 22.0 < scores ≤ 32.0. Severe: 32.0 < scores ≤ 100. The main points of comparison were subjective complaints, objective findings on corneal staining with fluorescein, meibomian glands and lid alterations and break-up time (BUT), basal secretion test, impression cytology of the conjunctival injection, and conjunctival staining. Subjects also underwent objective clinical assessment for DED.

### Statistical Analysis

Data were analyzed with the Statistical Package for Social Sciences version 24.0 software (SPSS Inc, Chicago, IL, USA). The descriptive statistics were presented as mean ± SD for normally distributed continuous variables. Nonnumerical data were recorded as presence (yes) or absence (no). Categorical variables were compared by using the Chi-square or Fisher exact test; continuous variables, using the one-way analysis of variance (ANOVA), Kruskal–Wallis test, and Jonckheere–Terpstra test. The Pearson Chi-square test and Fisher’s exact test were used for the rest of comparative and correlative analyses. Non-parametric Spearmen's correlation tests were used to determine differences between ocular surface *Demodex* infestation and other risk factors, and age-group analysis was performed using the Mantel–Haenszel test. Finally, Pearson correlation test and Mantel–Haenszel test were used to analyze the interaction between DM and DED in ocular *Demodex* infestation when graded. *P* < 0.05 was considered statistically significant in all analyses.

## Results

### Participants and the Basic Clinical Information

After applying all entry criteria, 444 eyes from 255 participants (aged 18–84) were included in the analysis. The missing data in the study are processed by the adjacent point interpolation method in SPSS software. Mean age was 53.96 (standard deviation [SD], 16.148) years. Overall, 45.1% of the participants were male, while 54.9% were female. Total OS *Demodex* infestation results were 23.4% for positive, 33.3% for suspicious positive, and 43.3% for negative.

In participants, there was a significant correlation between age and OS *Demodex* infestation (*P* < 0.001). We also found that OS *Demodex* infestation was strongly associated with MGD (*P* = 0.002), DED (*P* = 0.013), and OSDI scores (*P* = 0.030). Both DM (*P* < 0.001) and DM duration (*P* < 0.001) had significant correlation with OS *Demodex* infestation as well. Moreover, the prevalence of OS *Demodex* infestation was much higher in participants with hypertension (*P* = 0.016).

**Fig. 1:**
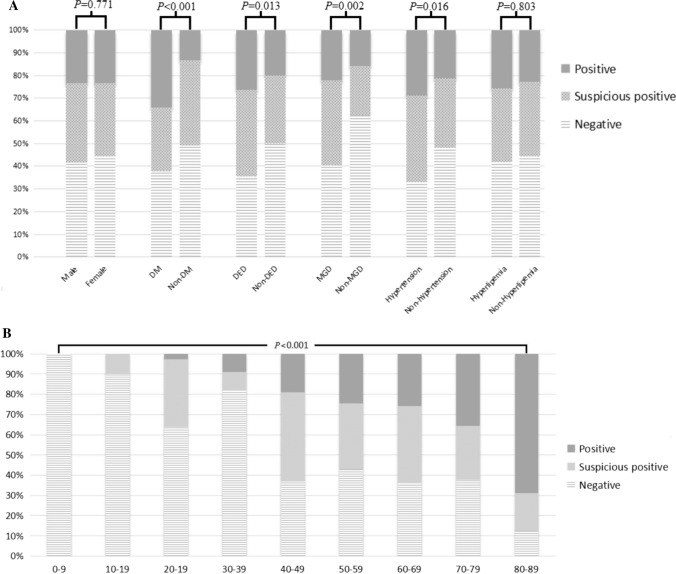
Differences between OS *Demodex* infestation and sex, DED, DM, hypertension, hyperlipidemia, MGD and age. **a** The relationship between ocular surface *Demodex* infestation and sex, diabetes mellitus, dry eye disease, meibomian gland dysfunction, hypertension and hyperlipidemia. After Chi-square test, diabetes mellitus, dry eye disease, tarsal gland dysfunction and hypertension were correlated with ocular surface *Demodex* infestation, but there was no statistical difference between sexes and hyperlipidemia. **b** The relationship between ocular surface *Demodex* infestation and age. After Jonckheere–Terpstra test, there was an apparent correlation between age and *Demodex* infestation, and a looming linear trend was also observed in the figure

However, no difference was exhibited between sex and OS *Demodex* infestation (*P* > 0.05), or between hyperlipidemia and OS *Demodex* infestation (*P* > 0.05). Further analysis of hematological parameters showed there was no difference between HbA1C, HDL, LDL, TG, TC, and OS *Demodex* infestation (Table 1; Fig. 1).

**Table 1:** Baseline characteristics of the participants, according to the infestation of OS *Demodex*

Characteristic	Negative	Suspicious positive	Positive	*P* value
Total *n* (%)	192 (43.2)	148 (33.3)	104 (23.4)	–
Age, (years)	49.84** ± **17.76	54.79** ± **4.13	62.12** ± **12.81	< 0.001^a^
Sex, (%)				
Male	41.6	35.0	23.4	0.771^b^
Female	44.5	32.0	23.5	
MGD, (%)				
Yes	40.8	36.7	22.4	0.002^b^
No	62.3	21.8	15.8	
DM, (%)				
Yes	37.9	28.0	34.1	< 0.001^b^
No	49.1	37.5	13.4	
DED, (%)				
Yes	35.5	38.1	26.3	0.013^b^
No	50.2	29.7	20.1	
Hypertension, (%)				
Yes	33.1	38.0	28.9	0.016^b^
No	48.2	30.7	21.1	
Hyperlipidemia, (%)				
Yes	41.9	32.4	25.7	0.803^b^
No	44.4	32.9	22.7	
DM duration (years)	2.56** ± **5.51	3.10** ± **5.91	6.34** ± **6.68	< 0.001^a^
HbA1C (%)	8.22** ± **2.89	8.26** ± **2.33	8.34** ± **2.72	0.586^a^
OSDI score	11.63** ± **11.23	14.88** ± **14.43	16.16** ± **16.65	0.030^a^
HDL (mg/dl)	1.27** ± **0.43	1.18** ± **0.29	1.32** ± **0.35	0.338^a^
LDL (mg/dl)	3.22** ± **0.96	2.86** ± **0.90	3.39** ± **1.24	0.681^a^
TG (mg/dl)	1.89** ± **1.91	1.96** ± **1.21	1.66** ± **1.26	0.927^a^
TC (mg/dl)	5.22** ± **2.27	4.90** ± **1.26	5.67** ± **2.59	0.173^a^

*DM* Diabetes mellitus, *DED* dry eye disease, *MGD* meibomian gland dysfunction, *HbA1C* glycosylated hemoglobin, *OSDI score* Ocular Surface Disease Index score, *HDL* high-density lipoprotein, *LDL* low-density lipoprotein, *TG* triglycerides, *TC* serum total cholesterol

^a^Jonckheere–Terpstra test

^b^Chi-square test

### The Relationship Between OS *Demodex* Infestation with DM and DED

From Table 1, both DM and DED had a significant correlation with OS *Demodex* infestation. We further divided DM into two layers (yes or no), and further statistically analyzed DED and OS *Demodex* infestation in each layer (Table 2), with results suggesting that there was no significant difference between DED and OS *Demodex* infestation in diabetic patients (*P* = 0.316), while in non-diabetic patients a difference between the latter two was distinguished (*P* = 0.017). Similarly, according to DED (yes or no), further statistical analysis of DM and OS *Demodex* infestation in each layer (Table 2) showed significant correlations between DM (yes or no) and OS *Demodex* infestation in patients with DED (DM: *P* = 0.015; Non-DM: *P* = 0.004).

**Table 2:** Relationship between DM, DED, and OS *Demodex* infestation

DM^a^	DED	Negative (%)	Suspicious positive (%)	Positive (%)	*P* value
Yes	Yes	34.5	29.9	35.6	0.316
	No	45.0	26.1	28.8	
No	Yes	36.4	45.5	18.2	0.017
	No	56.1	33.7	10.2	

DED^b^	DM	Negative (%)	Suspicious positive (%)	Positive (%)	*P* value
Yes	Yes	34.5	29.9	35.6	0.015
	No	36.4	45.5	18.2	
No	Yes	45.0	26.1	28.8	0.004
	No	56.1	33.7	10.2	

^a^Stratified analysis based on DM (yes or no): analysis of the relationship between DED and OS *Demodex* infestation at two levels

^b^Stratified analysis based on DED (yes or no): analysis of the relationship between DM and OS *Demodex* infestation at two levels

In short, both DM and DED had an nexus with OS *Demodex* infestation, and the association between DM and OS *Demodex* infestation was greater than DED. In other words, as long as the participants had diabetes, whether he/she had dry eye disease was insignificant for OS *Demodex* infestation.

Moreover, we divided all participants into four levels according to whether the patient had DM and DED: (1) DM- and DED- for the first level. (2) DM- and DED + for the second level. (3) DM + and DED- for the third level. (4) DM + and DED + for the fourth level. The prevalence and the statistical analysis of each group were shown in Table 3.

**Table 3:** Comprehensive analysis of the effects of DM and DED on OS *Demodex* infestation

OS *Demodex* infestation^a^	DM + and DED + (%)	DM + and DED- (%)	DM- and DED + (%)	DM- and DED- (%)	Pearson *χ*^2^ test	Pearson coefficient	
						*R*	*P*
−	17.5	29.2	21.1	32.2	< 0.001	0.197	< 0.001
±	19.5	21.8	33.8	24.8			
+	34.1	35.2	19.8	11.0			

^a^“ − ” refer to negative, “ ± ” refer to “suspicious positive”, “ + ” refer to “positive”

From grade 1 to grade 4, it was indicated that the results of OS *Demodex* infestation was becoming increasingly serious, and there was a significant linear positive correlation between them (M-H test: *P* < 0.001; Pearson coefficient: *R* = 0.197, *P* < 0.001). It indicated that both DM and DED were not only related to ocular surface *Demodex* infestation, but also played a synergistic role, which exacerbated the infestation of ocular surface *Demodex* together.

### Statistical Inference of Linear Trends Between Various Basic Indicators and Locust Infestation

In addition, Spearmen's correlation was used to analyze whether there was a significant linear correlation between ocular surface *Demodex* infestation and age (*ρ* = 0.265, *P* < 0.001), DM (*ρ* = 0.189, *P* < 0.001), DM duration (*ρ* = 0.256, *P* < 0.001). Also, there was a correlation between ocular surface *Demodex* infestation and MGD (*ρ* = 0.184, *P* < 0.001), DED (*ρ* = 0.138, *P* = 0.006 < 0.01), hypertension (*ρ* = 0.134, *P* = 0.05 < 0.01).

**Fig. 2:**
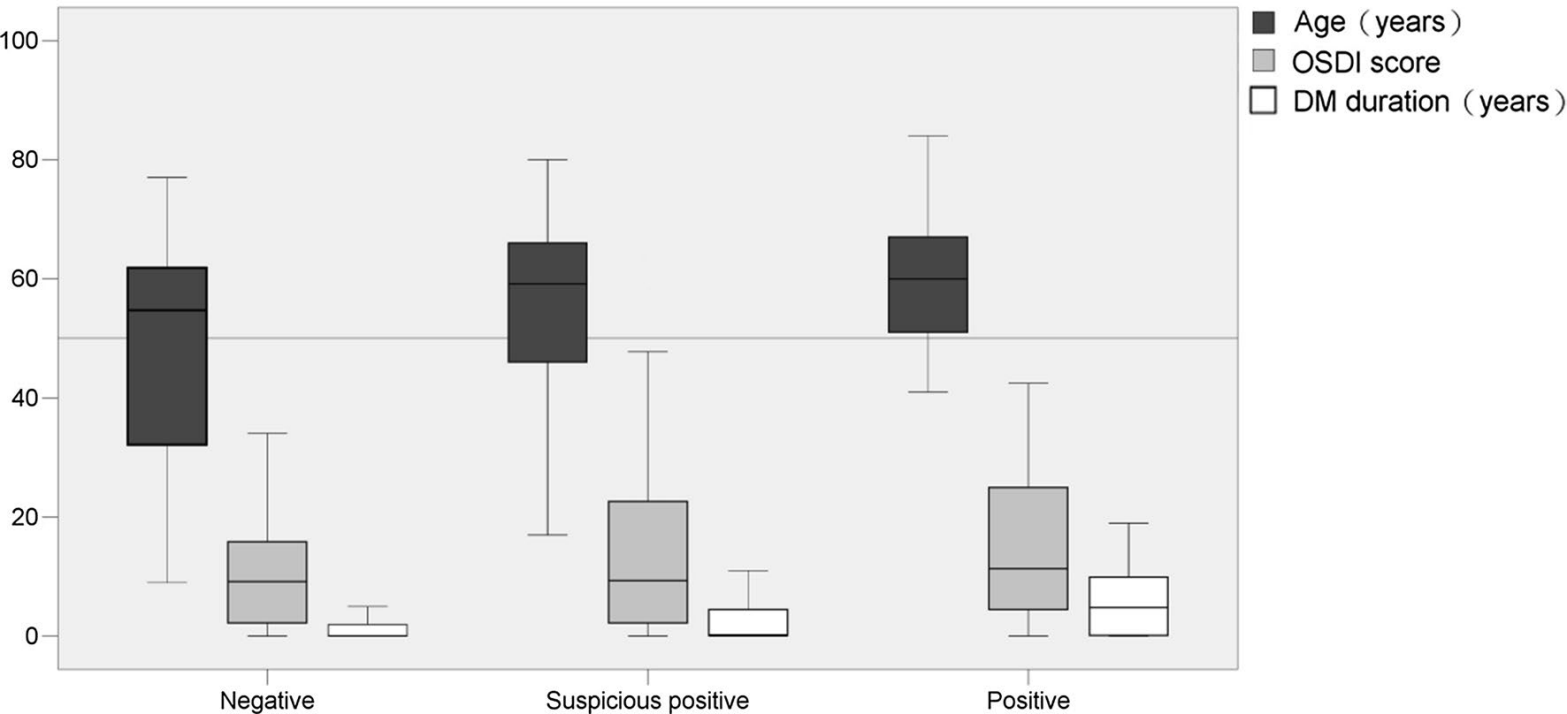
Further analysis of the age, DM duration, and OSDI scores in the OS *Demodex* infestation group by Box-plot. Age, OSDI score, and duration of diabetes increased with the severity of *Demodex* infestation, especially with age and OSDI score

From Table 1, we found an apparent correlation between age and OS *Demodex* infestation. After further grading the age, the severity of OS *Demodex* infestation is higher when the age increases, showing a linear trend at the same time, which suggested that age was a risk factor for OS *Demodex* infestation (Fig. 1). After further analyzed the relationship between age, OSDI score and DM duration and OS mite infestation, we discovered that age, OSDI score and duration of DM were positively correlated with the degree of OS *Demodex* infestation (Fig. 2; Table 4).

**Table 4:** Analysis of linear trends between OS *Demodex* infestation and age, DM duration, and OSDI scores

Characteristic	Pearson *χ*^2^ test		M-H test		Pearson coefficient	
	*χ*^2^ value	*P* value	*χ*^2^ value	*P* valule	*R* value	*P* value
Age	63.920	< 0.001	38.974	< 0.001	0.299	< 0.001
DM duration(year)	46.038	< 0.001	28.701	< 0.001	0.264	< 0.001
OSDI	6.459	0.596	1.558	0.212	0.069	0.212

*DM duration* Diabetic mellitus duration, *OSDI* Ocular Surface Disease Index

The results suggest a linear positive correlation between age and OS *Demodex* infestation (*R* = 0.299, *P* < 0.001), there was also a significant linear positive correlation (*R* = 0.264, *P* < 0.001) between the DM course and OS *Demodex* infestation, while the OSDI score was not significantly linearly correlated (*R* = 0.069, *P* > 0.05). It can be seen that with the increase of age, the infestation of OS *Demodex* gradually deteriorates, and as the length of DM is prolonged, the infestation of OS *Demodex* was also aggravated.

## Discussion

*Demodex*, a small parasitic mite that affects mammals, was first identified in 1841 [3]. However, only recently attracted the attention of clinicians, including ophthalmologists, dermatologists and other specialists [20] *Demodex* infestation rates increase with age, reaching 84% in the general population over 60 years of age and 100% in the general population over 70 years of age [20] There are a number of risk factors that may predispose patients to ocular vermiform, such as rosacea, alcohol intake, smoking, stress, and systemic immunocompromised status [1].

In recent years, some studies have shown that DM, DED, and OS *Demodex* infestation are related. However, there has not been a study to show whether the two diseases interact in the process of OS *Demodex* infestaion. To better understand it, we designed this study and demonstrated that DM and DED were both risk factors for OS *Demodex* infestation, and specifically uncovered that the association between DM and OS *Demodex* infestation had precedence over DED. Moreover, we also founded that patients with both diseases had higher risk of OS *Demodex* infestation.

Here we included 255 participants in our study. However, not all of the patients who participated in this study accepted all the projects involved in this study. For this part of the lost data, we adopt adjacent point interpolation method to make up for it. Fortunately, we found strong associations between OS *Demodex* infestation and age, MGD, DED, OSDI, DM, DM duration and hypertension. However, no notable correlation was found between OS *Demodex* infestation with hyperlipidemia. *Demodex* feeds on the accumulation of skin cells, hormones, and oils in hair follicles, so the gender of the host is thought to be related to the prevalence of ocular *Demodex.* Besides, Whereas Türk et al. reported that men had a higher rate of *Demodex* infestation than women [21]. However, our study supports that no discrepancy in OS *Demodex* infestation between men and women exists and many researchers [4, 5, 22] had the same results. Serological indicators including HbA1c, HDL, LDL, TG, and TC were also unrelated to OS *Demodex* infestation, indicating that serological indicators cannot be used as predictors of OS *Demodex* infestation.

There have been studies on the relationship between ocular surface *Demodex* infestation and diabetes mellitus. Keskin KR’s study found an increase in the density of *Demodex* in patients with gestational DM [16]. Yamashita LS’s found that *Demodex* infestations were more common in diabetic patients than in healthy volunteers [15]. Our research further certifies that DM is undoubtedly a risk factor of OS *Demodex* infes tation. DM has an adverse influence on the microvasculature in multiple organs, which aggravates OS *Demodex* infestation through biochemical pathways involved in facilitating and abrogating microvascular injury and reducing local mucosal reaction, which makes it easier to cause OS *Demodex* infestation [23].

Meanwhile, the close association between OS *Demodex* infestation and DED was observed in our research. Randon M’s study also suggested that *Demodex* was implicated in dry eye syndrome, which showed that in symptomatic patients, *Demodex* infestation was usually associated with MG dysfunction [19]. Initially, ocular surface *Demodex* infestations result in dry eye disease. Ocular *Demodex* can destroy the lacrimal glands and meibomian glands, which causes the reduction of tears and surface oils, leading to dry eye disease. However, because of the immunomodulatory function of tears on the ocular surface, DED causes a decrease in tear secretion, thus impairing the innate immune function of the ocular surface. This can lead to the occurrence of ocular surface *Demodex* infestation [24]. Overall, OS *Demodex* infestation and DED interact to jointly worsen ocular surface conditions.

The most essential finding of this study is that the relationship between DM and OS *Demodex* infestation is significantly greater than that between DED and OS *Demodex* infestation. After further analysis, we found that patients with both DM and DED had a higher severity of OS *Demodex* infestation than patients with only one disease, and it was significantly more dangerous than to those with neither disease, which suggests that there is a synergistic effect between DM and DED. The low local immunity of ocular surface may be the key to the interaction between DM and DED to aggravate ocular surface *Demodex* infestation. The high concentration of blood sugar in the body can cause inflammatory reaction on the ocular surface and damage the normal immune defense mechanism of the body. The occurrence of dry eye disease reduces tear secretion, which further weakens the intrinsic immune function of the ocular surface [23, 24].

Additionally, our study also reveals that age plays an important role in OS *Demodex* infestation. The analysis suggests a linear positive correlation between age and OS *Demodex* infestation. Therefore, we suggest that middle-aged and elderly people should take more notice of eye hygiene and the cleanliness of their living environment, so as to guard against OS *Demodex* infestation. Moreover, the prevalence of hypertension is also associated with OS *Demodex* infestation. The study showed that the severity of OS *Demodex* infestation in hypertensive patients was significantly higher than in people without hypertension. Vascular disease and micro-vascular hardening caused by hypertension, might resulting in the reduction of local immune function, blocking white blood cells (especially eosinophils), and consequently weakening anti-parasitic reactions, which worsens OS *Demodex* infestation [25].

In conclusion, our study successfully provides evidence that OS *Demodex* infestation is significantly associated with DM and DED, and DM plays a more significant role than DED in OS *Demodex* infestation. The severity of OS *Demodex* infestation in patients with both diseases was significantly higher than that in patients with only one disease and those without any disease. At the same time, age and hypertension have also been identified as risk factors for OS *Demodex* infestation. Overall, our research not only provides an evidence-based basis for clinical prevention of OS *Demodex* infestation, but also serves as a big reminder for the majority of people seeking healthy living.
